# The Age Factor in Ixekizumab Survival: Older Patients Show Higher Long-Term Treatment Survival

**DOI:** 10.3390/medicina61101827

**Published:** 2025-10-12

**Authors:** Inés Noval-Martín, Jorge Santos-Juanes, Irene Álvarez-Losada, Laura Palacios-García, Ana Lozano-Blazquez, Virginia García-Jimenez, Cristina Galache Osuna, Raquel Santos-Juanes Galache

**Affiliations:** 1 GRIDER—Grupo de Investigación en Dermatología, Universidad de Oviedo, 33006 Oviedo, Spain; inoval@hotmail.com (I.N.-M.); cristinagalache@gmail.com (C.G.O.); raquel.santosjuanes@gmail.com (R.S.-J.G.); 2 Unidad de Gestion Clínica de Dermatología, Hospital Universitario Central de Asturias, 33011 Oviedo, Spain; palaciossiero14@gmail.com; 3 Área de Dermatología, Departamento de Medicina, Universidad de Oviedo, 33006 Oviedo, Spain; 4 Instituto de Investigación Sanitaria del Principado de Asturias (ISPA), 33011 Oviedo, Spain; 5 Reumatology Department, Hospital Universitario de Santiago, 27004 Santiago de Compostela, Spain; irenealvarezlosada@gmail.com; 6 Unidad de Gestión Clínica de Farmacia, Hospital Universitario Central de Asturias, 33011 Oviedo, Spain; analozanob@icloud.com (A.L.-B.); virginia.garciaj@sespa.es (V.G.-J.)

**Keywords:** ixekizumab, real world, psoriasis

## Abstract

*Background and Objectives*: Ixekizumab is a human monoclonal antibody targeting interleukin-17A, approved for the treatment of moderate-to-severe plaque psoriasis. Given its demonstrated efficacy and safety in clinical trials, this study aimed to evaluate the real-world drug survival of Ixekizumab and identify clinical predictors of treatment discontinuation. *Materials and Methods*: A retrospective, observational, hospital-based study was conducted in the Department of Dermatology at the Central University Hospital of Asturias (HUCA). Patients with moderate-to-severe plaque psoriasis who initiated treatment with Ixekizumab (Taltz^®^) between 8 June 2017 and 10 October 2024, were included. Demographic data, comorbidities, age at disease onset, family history, PASI score, and previous treatments were recorded. Drug survival was assessed using Kaplan–Meier survival curves and the log-rank test. Predictors of discontinuation were analyzed using univariate and multivariate Cox proportional hazards models. *Results*: A total of 103 patients (55.3% women) were included. Drug survival rates were 85% at one year, 73% at two years, and 61% at four years, with a mean treatment duration of 52.5 months (95% CI: 46.01–58.99). Multivariate analysis showed that patients under the age of 65 had a significantly higher risk of treatment discontinuation (hazard ratio: 1.813; *p* < 0.05). The most common reason for discontinuation was secondary treatment failure (45.16%). Ixekizumab demonstrated sustained drug survival in a real-world setting, with rates falling within the mid-to-upper range reported in the literature. Older age (>65 years) was associated with greater treatment persistence, highlighting a potential influence of age on long-term therapeutic adherence.

## 1. Introduction

Psoriasis is a chronic, inflammatory, immune-mediated proliferative disease associated with T-cell dysfunction. It primarily affects the skin, nails, and joints, although it is now considered a systemic condition due to its frequent association with cardiovascular, metabolic, and neuropsychiatric comorbidities, among others [1,2]. Clinically, psoriasis is classified as mild, moderate, or severe. According to data from Europe and the United States, approximately 80% of patients present with mild forms of the disease. In moderate-to-severe cases, psoriasis has a significant impact on quality of life, with both physical and psychological consequences [1].

The most common clinical presentation of psoriasis is the so-called plaque psoriasis (psoriasis vulgaris), accounting for approximately 85–90% of all cases. It is characterized by well-demarcated erythematous plaques covered with whitish or silvery scales, typically distributed symmetrically. The most frequently affected sites include the scalp, elbows, and knees—particularly the extensor surfaces—as well as the umbilical area and sacral region, although lesions may appear on any part of the body. The disease follows a chronic course, marked by recurrent flares and periods of remission [3].

Current therapeutic options include topical treatments, systemic therapies, and phototherapy [4,5,6]. Treatment selection is determined by various factors, including clinical severity—typically classified as mild or moderate-to-severe—comorbidities, and the presence of psoriatic arthritis [6]. The introduction of biologic therapies has represented a major shift in the therapeutic goals for psoriasis. According to current guidelines, biologic agents may be used as first-line treatment for moderate-to-severe psoriasis, demonstrating greater efficacy compared to conventional systemic therapies such as methotrexate [7,8].

The pathophysiology of psoriasis involves a complex interplay between cytokines, specific receptors, and intracellular signaling molecules that ultimately result in an inflammatory cascade. Biologic therapies act by selectively targeting key molecules involved in the disease process. The most relevant signaling pathways in plaque psoriasis include tumor necrosis factor-alpha (TNF-α), interleukin-23 (IL-23), and interleukin-17 (IL-17) [9]. The IL-17 family comprises structurally related cytokines, from IL-17A to IL-17F. Among these, IL-17A, IL-17C, and IL-17F are most prominently expressed in psoriatic lesions. IL-17A, secreted by activated T-helper cells, acts primarily on keratinocytes, stimulating the production of proinflammatory cytokines, chemokines, and other mediators that recruit neutrophils, macrophages, and lymphocytes, and activate local fibroblasts. Furthermore, IL-17 has been linked to increased keratinocyte proliferation and mitotic activity [9].

Ixekizumab is a humanized IgG4 monoclonal antibody that selectively binds to IL-17A with high specificity and affinity thereby neutralizing its biological activity and disrupting the proinflammatory cascade typical of psoriasis [10,11]. Although IL-17C and IL-17F are found at higher concentrations in psoriatic lesions, IL-17A is considered the most biologically active cytokine of the group and a central mediator of inflammation and tissue damage in plaque psoriasis [9]. Ixekizumab was approved by the U.S. Food and Drug Administration (FDA) in March 2016, and subsequently by the European Medicines Agency (EMA), for the treatment of moderate-to-severe plaque psoriasis in adult patients who are candidates for systemic therapy or phototherapy. The recommended dosing regimen includes a 160 mg initial dose administered subcutaneously (as two 80 mg injections) at week 0, followed by 80 mg every two weeks through week 12. From week 16 onwards, a maintenance dose of 80 mg every four weeks is indicated [12,13].

Drug survival refers to the duration over which a treatment remains effective, safe, and acceptable to the patient [14]. This metric not only reflects therapeutic effectiveness and tolerability but also encompasses patient satisfaction and adherence. Given that drug survival may be influenced by both clinical efficacy and patient behavior (i.e., adherence), future studies should incorporate adherence measures to provide a more accurate assessment of real-world treatment outcomes. This consideration is particularly relevant, as non-adherence can confound the interpretation of drug survival data and lead to overly optimistic estimates of treatment effectiveness and safety [15].

The aim of this study was to assess the long-term drug survival of ixekizumab and to identify clinical predictors of treatment discontinuation in patients with plaque psoriasis managed at the Dermatology Department of HUCA.

## 2. Materials and Methods

We performed a retrospective, single-center study in the Department of Dermatology at the Central University Hospital of Asturias (HUCA), including patients treated with ixekizumab between 8 June 2017, and 10 October 2024. The study protocol was approved by the Ethics and Research Committee of the Principality of Asturias, Spain (Ref. 2024-432).

A total of 103 patients who received standard doses of ixekizumab (Taltz^®^) for plaque psoriasis were included. Clinical and demographic baseline data were retrieved from electronic medical records. The following variables were collected: sex, age, weight, height, family history of psoriasis (at least one affected first-degree relative), age at disease onset, prior exposure to biologics (categorized as biologic-naïve or previously treated), and presence of psoriatic arthritis confirmed by a rheumatologist.

Comorbidities such as hypertension, diabetes mellitus, and dyslipidemia were also recorded. These were identified through medical history, patient report, or active treatment with antihypertensive, antidiabetic, or lipid-lowering agents. Patients with systolic/diastolic blood pressure > 135/85 mmHg at consultation were also classified as hypertensive. Dyslipidemia was defined as triglycerides > 150 mg/dL, total cholesterol > 200 mg/dL, or LDL cholesterol > 160 mg/dL. Body mass index (BMI) was calculated as weight in kilograms divided by height squared in meters (kg/m^2^), with obesity defined as BMI ≥ 30 kg/m^2^, following World Health Organization (WHO) criteria.

Drug survival was defined as the time from treatment initiation to permanent discontinuation. Treatment response was classified as primary failure when PASI 75 was not achieved at week 16, and as secondary failure when loss of PASI 75 occurred after an initial response beyond week 16. Uncontrolled joint disease was defined as persistence of prior symptoms or new-onset arthritis during follow-up.

### Statistical Analysis

Data were summarised and statistical analyses performed with IBM SPSS version 27.0 (IBM Corp., Armonk, NY, USA). Data are presented as the mean ± standard deviation for continuous variables, and the number and percentage for categorical variables. Group differences for qualitative variables were investigated with the chi-square test. Survival curves were derived using the Kaplan–Meier estimator and compared using the long-rank test. Cox proportional hazard regression models were used for multivariate analyses, and unadjusted and adjusted hazard ratios (HR) were both used to summarize the studied differences. 95% confidence intervals (95% CIs) are also provided. The proportionality of the risks was checked beforehand using the Schoenfeld residual.

We selected the following variables as possible predictors: sex, age of onset of psoriasis, age at treatment initiation, family history, presence of obesity, arthritis, arterial hypertension, diabetes and dyslipidemia, and previous use of biological drugs (>1). Group differences were considered statistically significant for values of *p* < 0.05.

The final sample size was sufficient to enable hazard ratios >1.75, proportional differences >25% and standardized differences in means >0.5 to be considered significant (Type I error = 0.05, Type II error = 0.2).

## 3. Results

### 3.1. Patient Characteristics

The study cohort comprised 103 patients, all identified as of White ethnicity. Baseline characteristics are detailed in Table 1. The differences between the two groups are that patients aged ≥ 65 years had a higher age at treatment initiation, a later age at psoriasis onset, a longer mean duration of ixekizumab therapy, and a higher prevalence of comorbidities such as hypertension and dyslipidemia.

### 3.2. Drug Survival

**Figure 1:**
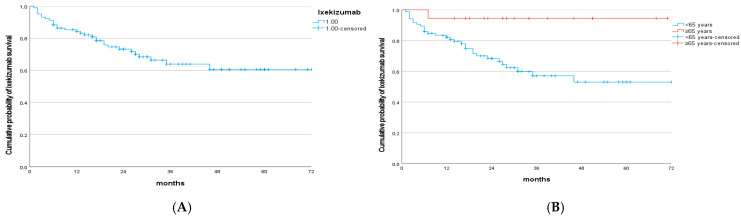
(**A**) Kaplan–Meier curve of ixekizumab survival. (**B**) Kaplan–Meier curve of ixekizumab survival according to age.

The survival of the drug is shown in Figure 1.

Overall ixekizumab survival was 85% at year 1, 73% at year 2, and 61% from year 4 through year 6. The mean drug survival was 52.5 months (95% CI, 46.01–58.99). Using the log-rank test, statistically significant differences were observed according to treatment initiation at age < 65 years (*p* = 0.014) (Figure 1B). However, no significant differences in drug survival were found with respect to the presence of arthritis (*p* = 0.509), obesity (*p* = 0.441), prior biologic exposure (*p* = 0.868), or female sex (*p* = 0.139). Likewise, no significant differences were observed for hypertension (*p* = 0.680) or dyslipidemia (*p* = 868).

### 3.3. Univariate Analysis

In the univariate analysis, statistically significant differences were observed only for age at treatment initiation with ixekizumab (≥65 years; *p* = 0.039). Patients who started treatment at ≥65 years had a significantly lower risk of discontinuation compared with those who initiated therapy before the age of 65 (HR = 0.123; 95% CI, 0.017–0.902), indicating that discontinuation was more frequent among younger patients (Table 2).

### 3.4. Adverse Effects

Adverse events were recorded in seven patients, including oral aphthae, urticaria, diarrhea (three cases), recurrent oropharyngeal candidiasis, and vitiligo. In addition, two deaths were reported, attributable to hepatic cirrhosis and oropharyngeal carcinoma, respectively.

### 3.5. Patients Discontinuing Treatment

At the end of the study, 72 out of the 103 patients who initiated treatment with ixekizumab remained on therapy (69.9%). A total of 31 patients (30.1%) discontinued treatment due to the following reasons: primary failure in 8 patients (25.80%), secondary failure in 14 patients (45.16%), the occurrence of adverse events in 7 patients (22.58%) and during follow-up two deaths occurred, both unrelated to the ixekizumab: oropharyngeal carcinoma and the other to liver cirrhosis (6.45%). In the group aged >65 years, only one patient discontinued treatment due to secondary failure.

## 4. Discussion

In this study, we report a series of patients treated with ixekizumab in a real-world clinical practice setting. The interpretation of our findings must consider the inherent difficulty of comparing results across studies, largely due to heterogeneity in methodological approaches, differences in publication timelines, and variability in the clinical variables included in each analysis. Such factors represent significant challenges for drawing robust, generalizable conclusions. Indeed, the degree of variability can be so pronounced that, even within the same study, drug survival has been shown to differ between recruiting hospital centers [16].

Although numerous studies have investigated ixekizumab use in real-world settings [17,18,19,20,21,22,23,24,25,26,27,28,29,30,31,32,33,34,35,36,37,38,39,40] relatively few have specifically evaluated treatment survival [41,42,43,44,45,46,47,48,49,50,51,52,53,54]. This underscores the relevance of our work, as it provides additional evidence on long-term persistence and the reasons for discontinuation, contributing to a better understanding of ixekizumab performance beyond clinical trial conditions.

The demographic characteristics of our cohort are comparable to those described in a review of studies published between 2016 and 2021 [10]. The mean age of our patients was 51.1 years, within the range reported in that review, where mean ages varied between 45 and 53.6 years. These findings are also consistent with those published by Caldarola et al. [18], Gargiulo [53], and Gottlieb [24]. In our clinical practice, the mean age of patients treated with ixekizumab was slightly higher than that observed in our previous series with other biologic agents, such as ustekinumab (47.9 years) [55], adalimumab (45.9 years) [56], and secukinumab (50.5 years) [57]. In our cohort, women accounted for the majority of patients (55.3%), a finding that contrasts with most published series, in which male predominance has been consistently reported [20,22,48,53].

A distinctive feature of our cohort is the remarkably high proportion of patients with prior exposure to biologic therapies (96.1%), substantially exceeding the figures reported in previously published studies: Caldarola (56.86%) [18], Gargiulo (51.7%) [53], Gottlieb (48.6%) [24], and Fitzgerald (58.2%) [54]. This finding is largely explained by the therapeutic policy currently implemented in our region. According to the protocol established by the Commission for the Rational Use of Medicines of the Principality of Asturias (CURMP), adalimumab biosimilars are prioritized as the first-line biologic option for moderate-to-severe plaque psoriasis. Consequently, patients eligible for subsequent lines of therapy, such as ixekizumab, are predominantly biologic-experienced, which may influence both treatment survival and the comparability of our results with those of other cohorts.

In our cohort, the probability of continuing treatment with ixekizumab was 85% at one year, 73% at two years, and 61% between the fourth and sixth years. These data reflect a high drug survival during the first year, although slightly lower than that reported in some studies, such as Caldarola (92.11%) [18] and Gargiulo (95.6%) [53]. Our results are comparable to those of Ting (87.2%) [58] and Lockshin (81%) [48], and considerably higher than those reported by Zhdanava (63.4% at one year) [31]. At four years of follow-up, our data show a survival rate slightly lower than that described by Torres (71%) [51]. At five years, our findings are similar to those reported by Ting (59.4%) [58] and Mastorino, who observed a continuation rate of 66% [22]. However, our four-year rates are considerably lower than those reported by Gargiulo, who documented a survival of 82.6% at the same time point [53]. These differences at four years may be attributed to the high proportion of patients with prior exposure to biologic therapies in our cohort. In the literature, there is no clear consensus regarding the impact of previous biologic use on drug survival. However, several studies suggest a potential association, both for biologic agents in general [20] and specifically for the interleukin-17 inhibitor class [50]. In particular, the study by Gargiulo highlights this factor as relevant to the long-term continuation of ixekizumab treatment [53].

One factor that may have positively contributed to treatment adherence in our cohort is the change in ixekizumab formulation from citrate-containing to citrate-free. This new formulation was preferred and well tolerated by most patients who switched from the original presentation, which may have facilitated treatment continuation. Similarly, patients who initiated therapy directly with the citrate-free formulation also showed good acceptance, consistent with the findings reported by Chabra et al. [59]. These observations suggest that factors such as formulation tolerability may play a relevant role in long-term drug survival, beyond the clinical and demographic variables traditionally considered.

In our study, no patient clinical or demographic variables, nor disease-related characteristics, were found to significantly influence ixekizumab drug survival, with the exception of older age. Specifically, gender showed no association with treatment discontinuation, in line with the findings of Schots [49] and Malagoli [60], but in contrast with those of Graier, who reported a higher risk of discontinuation in women [50].

Regarding prior exposure to biologic therapies, our analysis specifically compared patients with exposure to a single biologic versus those who had received multiple biologic treatments. Although several studies have demonstrated that prior biologic exposure is associated with an increased risk of discontinuation [18,20,50,51,53], our results are consistent with other reports showing no significant influence of this factor [43,49,60]. In our analysis, obesity was not associated with an increased likelihood of treatment discontinuation. However, Caldarola et al. [18] reported that a body weight ≥ 90 kg was linked to reduced drug survival. Similarly, Mastorino [22] and Torres [51] observed decreased treatment effectiveness in patients with higher mean BMI values.

Taken together, these discrepancies highlight the lack of consensus in the literature and underscore the need for larger, multicenter studies with stratified analyses to better elucidate the impact of demographic, clinical, and therapeutic variables on long-term drug survival.

However, statistically significant differences were observed in the univariate analysis with respect to age, showing greater treatment survival in patients who initiated ixekizumab at ≥65 years. This finding contrasts with the results of a previous study from our group, in which other biologic treatments—but not ixekizumab, due to the very small number of patients—showed higher survival in older patients without reaching statistical significance [61], similar to what has been more recently reported [62]. We were unable to identify a clear explanation for the higher drug survival observed in patients aged >65 years. Although previous studies have suggested that biologics may be more frequently prescribed in elderly patients with fewer comorbidities, in our cohort patients aged >65 years actually presented a higher prevalence of all comorbidities. We also considered the potential influence of treatment behavior on drug survival. Older patients may show greater compliance and disease awareness, partly due to their experience managing multiple chronic conditions that require strict adherence to therapy. This behavioral factor, although not directly determined by age itself, could indirectly enhance treatment persistence and may help explain the higher survival observed in patients aged ≥65 years in our cohort.It should be noted that the observed effect of age (≥65 years) on treatment survival is based on a small subgroup (*n* = 18) with wide confidence intervals. Therefore, this finding should be interpreted as exploratory rather than definitive.

Of the 103 patients who initiated treatment with ixekizumab, 31 discontinued therapies during follow-up (30.1%). The overall discontinuation rate in our cohort was slightly higher than that reported in studies by Caldarola (22.54%) [18] and Lockshin (22.00%) [25]. With regard to discontinuations due to adverse events, our series showed a rate of 7%, which is consistent with the findings of Chiricozzi (4%) [36] and Malagoli (6%) [60], but markedly lower than the rate reported by Mastorino (32.8%) [22]. This variability may reflect differences in patient selection, safety monitoring, and reporting criteria across studies.

Our study has several limitations that should be considered when interpreting the results. First, it is a retrospective study and therefore subject to the inherent biases of this design. Second, the study was conducted in a single center, which may also limit the generalizability of the results. Third, treatment selection was not randomized but rather based on individual clinical criteria in the context of routine practice, which may introduce selection bias. Fourth, the sample size was limited, and a substantial proportion of patients were still receiving treatment at the study cutoff date, which could affect estimates of drug survival. Fifth, the recent availability of new biologic therapies may have led to a greater tendency to switch treatments after shorter treatment periods in recent years. Sixth, the small sample size in the subgroup of patients older than 65 years may limit the robustness of the results, and therefore these findings must be taken with caution. Finally, the concomitant use of topical therapies was not accounted for in the analysis. This study provides real-world evidence on ixekizumab use with up to six years of follow-up, contributing information beyond the controlled setting of clinical trials. The systematic collection of data within a single tertiary hospital ensured consistency in patient management and follow-up. In addition, the analysis of drug survival and reasons for discontinuation offers insights that may support clinical decision-making and health policy.

## 5. Conclusions

In conclusion, our study shows that ixekizumab, in a cohort with a high proportion of non-naïve patients, demonstrates drug survival rates within the upper range of those reported in the literature. Survival was higher among patients aged over 65 years. This finding, which has not been previously described, suggests that ixekizumab is an effective long-term treatment option even in heavily pretreated populations. Nonetheless, Future studies should aim to validate these findings in larger, multicenter cohorts and explore predictive factors of treatment persistence, including clinical characteristics, biomarkers, and comorbidities. Comparative analyses with other biologics and evaluations of long-term safety and quality-of-life outcomes would also provide valuable insights.

## Figures and Tables

**Table 1:** Baseline characteristics of patients.

	Total Patients (*n* = 103)	<65 y (*n* = 85)	≥65 y (*n* = 18)	*p*
Sex (male), *n* (%)	46 (44.7%)	39 (45.9%)	7 (38.9%)	0.779
Age at start of biologic treatment (years), mean ± SD	51.1 ± 14.3	46.85 ± 11.76	71.22 ± 4.58	0.001
Positive family history of psoriasis (yes), *n* (%)	67 (65%)	58 (68.2%)	9 (50%)	0.229
Onset before 40 years of age (%)	82 (79.6%)	75 (88.2%)	11 (61.1%)	0.014
Duration of treatment (months); mean	52.5 (IC95% = 46.01–58.99)	48.30 (IC = 40.89–55.71)	67.44 (IC = 60.67–74.20)	0.003
Initial PASI	13.2 ± 6.1	14.4 ± 7.3	12.8 ± 5.8	0.32
Comorbidities, n (%)				
Obesity (BMI ≥ 30)	47 (45.6%)	41 (48.2%)	6 (33.3)	0.372
Diabetes mellitus	19 (18.4%)	13 (15.3%)	6 (33.3%)	0.145
Arterial hypertension	46 (44.7%)	33 (38.8%)	13 (72.2%)	0.020
Dyslipidemia	44 (42.7%)	32 (37.6%)	12 (66.7%)	0.046
Arthritis	59 (57.3%)	49 (57.6)	10 (55.6%)	1.000
Prior treatments with biologics (%)	99 (96.1%)	82 (96.5%)	17 (94.4%)	1.000
One biologic	37 (35.9%)	30 (35.3%)	7 (38.9%)	
Two biologics	39 (37.9%)	33 (38.8%)	6 (33.3%)	0.115
Three biologics	16 (15.5%)	15 (17.6%)	1(5.6%)	
Four biologics	6 (5.8%)	4 (4.7%)	2 (11.1%)	
Five Biologics	1 (1%)	0	1 (5.6%)	

**Table 2:** Univariate and multivariate analysis of predictors of discontinuation with *p*-values and 95% confidence intervals.

	Univariate Analysis		Multivariate Analysis	
Univariate Analysis	*p*-Value	HR (95% CI)	*p*-Value	HR (95% CI)
Age at treatment initiation ≥ 65 years	0.039	0.123 (0.017–0.902)	0.023	0.079 (0.009–0.700)
Age at psoriasis onset ≥ 40 years	0.238	0.986 (0.964–1.009)	0.534	1.475 (0.432–5.034)
Female sex	0.145	1.729 (0.827–3.612)	0.054	2.436 (0.986–6.019)
Obesity (BMI ≥ 30)	0.444	1.317 (0.650–2.667)	0.546	1.271 (0.583–2775)
Arthritis (yes)	0.512	0.789 (0.388–1.604)	0.287	0.654 (0.299–1.431)
Hypertension (yes)	0.277	0.671 (0.327–1.377)	0.578	0.774 (0.313–1.913)
Dyslipidemia (yes)	0.540	1.248 (0.615–2.529)	0.104	1.975 (0.869–4.489)
Family history of psoriasis (yes)	0.591	1.230 (0.578–2.616)	0.762	0.880 (0.383–2.019)
Diabetes (yes)	0.868	0.927 (0.380–2.262)	0.796	0.869 (0.299–2.524)
>1 prior biologic	0.869	0.939 (0.445–1.983)	0.813	1.105 (0.481–2.019)

HR: hazard ratio; CI: confidence interval; BMI: body mass index.

## Data Availability

The data presented in this study are available upon request from the corresponding author.
